# The experiences, attitudes and understanding of research amongst medical students at an Australian medical school

**DOI:** 10.1186/s12909-021-02713-9

**Published:** 2021-05-10

**Authors:** Jaidyn Muhandiramge, Tony Vu, Megan J. Wallace, Eva Segelov

**Affiliations:** 1 School of Medicine, Faculty of Medicine, Nursing and Health Sciences, Monash University, Melbourne, Australia; 2 Department of Obstetrics and Gynaecology, Monash University, Melbourne, Australia; 3 School of Clinical Sciences, Monash University, Melbourne, Australia

**Keywords:** Medical student research, Research education, Research motivation, Research barriers, Research attitudes, Research experiences, Clinician-scientist, Physician-scientist, MD, Research training

## Abstract

**Background:**

Research engagement plays an integral role in developing clinicians that practice effective, evidence-based medicine. Research participation by clinicians, however, is declining. Given the link between research during medical school and future research output, promotion of medical student research is one avenue by which this shortage can be addressed. Student research attitudes and participation in Australia are not well-documented in the literature. This study therefore aims to investigate research practices, motivators, and barriers amongst Australian medical students in order to determine whether there is a need for further integration of research within Australian medical school curriculums.

**Methods:**

A cross-sectional study design was used to explore research experience and attitudes, as well as the enablers and barriers to research amongst students enrolled in all years of the five-year medical course at Monash University. A questionnaire was created by combining questions from several surveys on medical student research and comprised Likert scales, multiple choice options and free-text responses assessing research experience, attitudes, motivators, and barriers.

**Results:**

Seven hundred and four respondents (69.4% female; survey response rate 36.7%) reported variable research experience and interest. Less than half of the cohort (*n* = 296; 44.9%) had contributed to a research project. Increasing employability for specialty training programs was the primary motivating factor (*n* = 345; 51.9%) for pursuing research, with only 20.5% (*n* = 136) citing an interest in academia as a motivator. Time constraints (*n* = 460; 65.3%) and uncertainty surrounding how to find research opportunities (*n* = 449; 63.8%) were the most common barriers to research.

**Conclusions:**

Medical students at Monash University are interested in but have limited experience with research. Students are, however, primarily motivated by the prospect of increasing employability for specialist training; medical schools should therefore focus on encouraging intrinsic motivation for pursuing research. Greater integration of research education and opportunities within medical school curricula may also be required to provide students with the skills necessary to both pursue research and practice evidence-based medicine.

**Supplementary Information:**

The online version contains supplementary material available at 10.1186/s12909-021-02713-9.

## Background

The rapid advancement of medical research and technology in the twenty-first century has seen evidence-based medicine become the standard for clinical practice. Research participation plays a key role in the development of skills that help clinicians practice evidence-based medicine. Research engagement amongst clinicians, however, appears to be in decline [1, 2]. Since its peak in the 1980s, the proportion of physicians in the United States actively participating in research has decreased from 4.7% of the total clinical workforce to 1.5% today [3].

A significant contributor to this shortage is thought to stem from limited research engagement by medical students [4, 5], especially given that clinicians who have conducted research in medical school typically have greater postgraduate research output [6]. Research participation additionally provides numerous benefits to clinicians beyond improving the practice of evidence-based medicine, including problem solving, critical thinking, and literature interpretation [1, 2]. The meaningfulness of medical student contributions should also not be overlooked; Jay McLean’s discovery of heparin, Paul Langerhans’ description of pancreatic islets, and Martin Flack’s discovery of the sinoatrial node are a few of many notable contributions made by students while studying medicine [7].

Australian medical schools currently offer both direct-entry and graduate-entry courses, with course duration ranging from four to 6 years. The emphasis on research within the standard curriculum of the 21 universities offering a medical degree varies; some include a research project of variable length as part of the medical curriculum, while others allow the intercalation of a research degree [8]. Only five, however, offer a combined Doctor of Medicine/Doctor of Philosophy (MD-PhD) program, compared to 90 of 153 schools in the United States [9].

There are limited recent studies in the Australian medical school setting exploring students’ research practices and attitudes and as a result, little is known about Australian medical student research within the context of future aspirations [5, 10, 11]. The rapid move by Australian universities to more research-oriented Doctor of Medicine degrees, along with the pressure on universities to compete internationally with increasing research outputs, further necessitates investigation of Australian medical student research. This study therefore asked the following research questions: 1) what are the attitudes of Australian medical students towards research, 2) what experiences have medical students had with research, and 3) what are the enablers and barriers to research amongst Australian medical students?

## Methods

Ethics approval was received from the Monash University Human Research Ethics Committee (Project ID 18400).

### Study cohort

Monash University offers direct-from-school medical training (5 years) at their Australian and Malaysian campuses and graduate-entry medical training (4 years) after completing a biomedical science, pharmacy, physiotherapy, or science degree at their Australian campuses. Clinical training is distributed across metropolitan Melbourne (17 sites) and rural Victoria (13 sites), and in two sites in Malaysia. The degree includes basic research education covering introductory level epidemiology and biostatistics, but there is no compulsory requirement for practical research involvement. However, students can opt to undertake an intercalated one-year Bachelor of Medical Science (Honours) (BMedSc (Hons)) degree (65–80 students/year) and a small number continue directly onto a Doctor of Philosophy (PhD) (3–5 students/year).

The postgraduate Monash medical student cohort is largely composed of students who have previously completed a Bachelor of Biomedical Science, a degree with limited practical research exposure but some basic teaching on epidemiology and biostatistics. The degree also features a capstone unit in its final year which requires students to write a systematic review.

### Survey

Data was collected via a questionnaire developed on the Qualtrics XM® platform. A 28-point survey, consisting of qualitative (free-text responses) and quantitative questions (multiple-choice questions, checkbox questions, Likert scales) was developed (see Additional file 1) combining questions from previous studies investigating medical student research following an extensive review of the literature. Five questions were optional. The questions assessed participant characteristics as well as research experience, attitudes, motivators, and barriers. For most participants, survey responses represent experiences during enrolment in the medical program; however, for the small group of graduate-entry students, some responses may reflect experiences from their undergraduate degree. The survey was piloted with ten students to refine comprehensibility.

The survey was distributed through Moodle (Monash University’s learning management system) and the official Monash University medical student Facebook groups in July 2019. Posts were made at the start of the month-long survey period, with reminders at two and four weeks. Respondents were offered entry into a draw for four $50 gift cards.

### Data analysis

Data was stratified using the cross-tabulation feature in Qualtrics XM®. SPSS Statistics 26® was used to analyse quantitative data using Pearson’s chi-squared tests. Cramer’s V test was used to calculate effect size. A *p* value < 0.05 was considered as statistically significant. Free-text responses to short answer questions were analysed individually by the investigators and sorted into common and recurring themes. Figures were created using GraphPad Prism 8®. A difference of < 5% between groups was reported as no difference.

## Results

Seven hundred and four responses were received from the 2019 total enrolment of 1917 students at all Australian sites, yielding a response rate of 36.7%. Seventy-four responses were incomplete but were included in analysis. Demographics of respondents are presented in Table 1.

**Table 1:** Demographics of survey respondents

	Respondents, n (%)
**Gender** (%)	
Male	186 (29.5)
Female	437 (69.4)
Other	7 (1.1)
**Age (years)** (%)	
18–21	382 (54.5)
22–25	277 (39.5)
26–29	32 (4.6)
30+	10 (1.4)
**Type of enrolment** (%)	
Direct-entry	568 (79.3)
Graduate-entry	146 (20.7)
**Year level** (%)	
1	133 (18.9)
2^a^	169 (24.0)
3	117 (16.6)
4	137 (19.5)
5	109 (15.5)
Bachelor of Medical Science (Honours)	35 (5.0)
PhD	4 (0.6)
**Type of medical place** (%)	
Commonwealth Supported Place	362 (51.4)
Bonded Medical Place	136 (19.3)
Extended Rural Cohort	53 (7.5)
Full Fee Paying	153 (21.7)

^a^ Direct-entry students are referred to as years one through to five, whereas graduate-entry students are referred to as years A through to D. For presentation purposes, direct-entry and graduate-entry students were grouped together by their relevant year level (e.g. 2nd year direct-entry and 1st year graduate-entry students [Year A] at Monash University are both labelled as “Year 2”)

While the Monash medical school cohort is predominantly female (56.6%), an even greater proportion of survey respondents were female. The type of enrolment and year level splits amongst the survey cohort was roughly similar to that of the Monash medical school cohort, where approximately 20.9% of students are graduate entry and year level sizes are approximately similar.

### Research experience

**Fig. 1:**
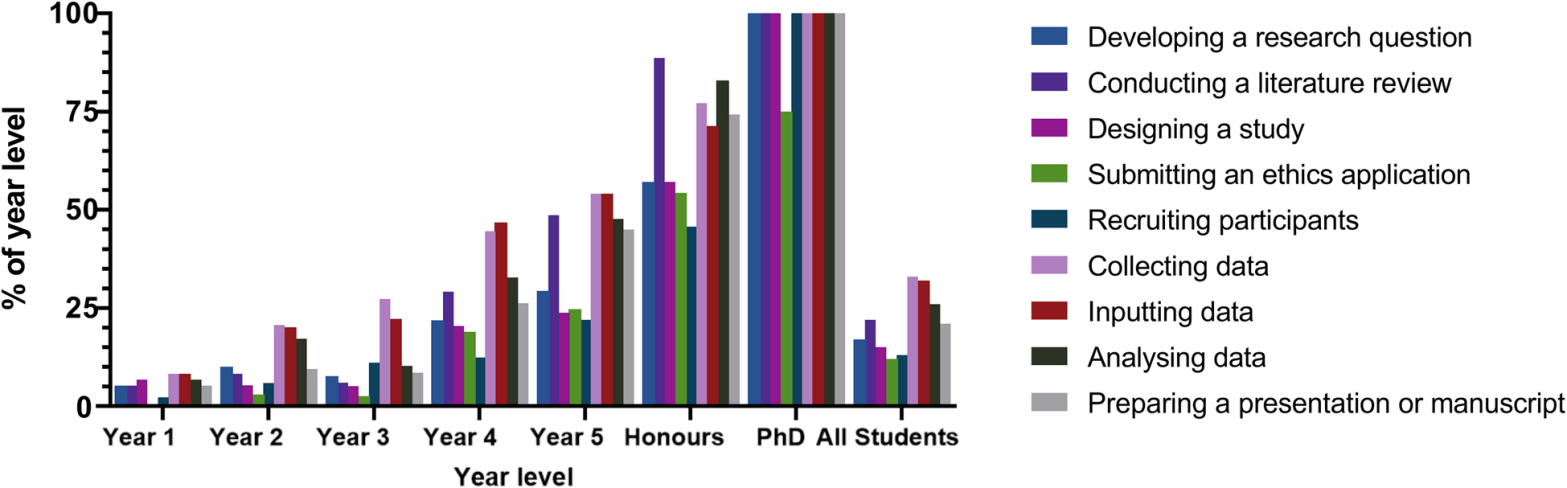
Research tasks completed by students, stratified by year level

The majority of respondents (*n* = 363; 55.1%) had not been involved in research in any capacity, while 236 students (36.0%) had been involved in one-to-three projects and 58 students (9.0%) reported involvement in four-or-more projects.^1^ The types of contributions to projects varied by year level (Fig. 1). Graduate-entry students reported greater research experience than direct-entry students (*n* = 85; 63.9% versus *n* = 209; 39.9%), as well as greater contributions in all domains except for participant recruitment. There was no difference between genders in research output (data not shown).

Of the 296 students involved in research, 119 students (40.9%; 17.2% of the total study cohort) had been named an author on an output of a project, 23.3% (*n* = 69; 9.8% of the total study cohort) had been published in a peer-reviewed journal, 17.2% (*n* = 51; 7.2% of the total study cohort) had presented at national conferences, and 12.8% (*n* = 38; 5.4% of total study cohort) had presented internationally. Authorship in any capacity generally increased with advancing year level and did not vary with gender (data not shown). While students interested in medical/physician specialities, surgical specialities, and obstetrics and gynaecology reported the greatest rates of authorship, only the latter yielded a significant association (χ2(4) = 10.838, *p* < 0.05).

**Fig. 2:**
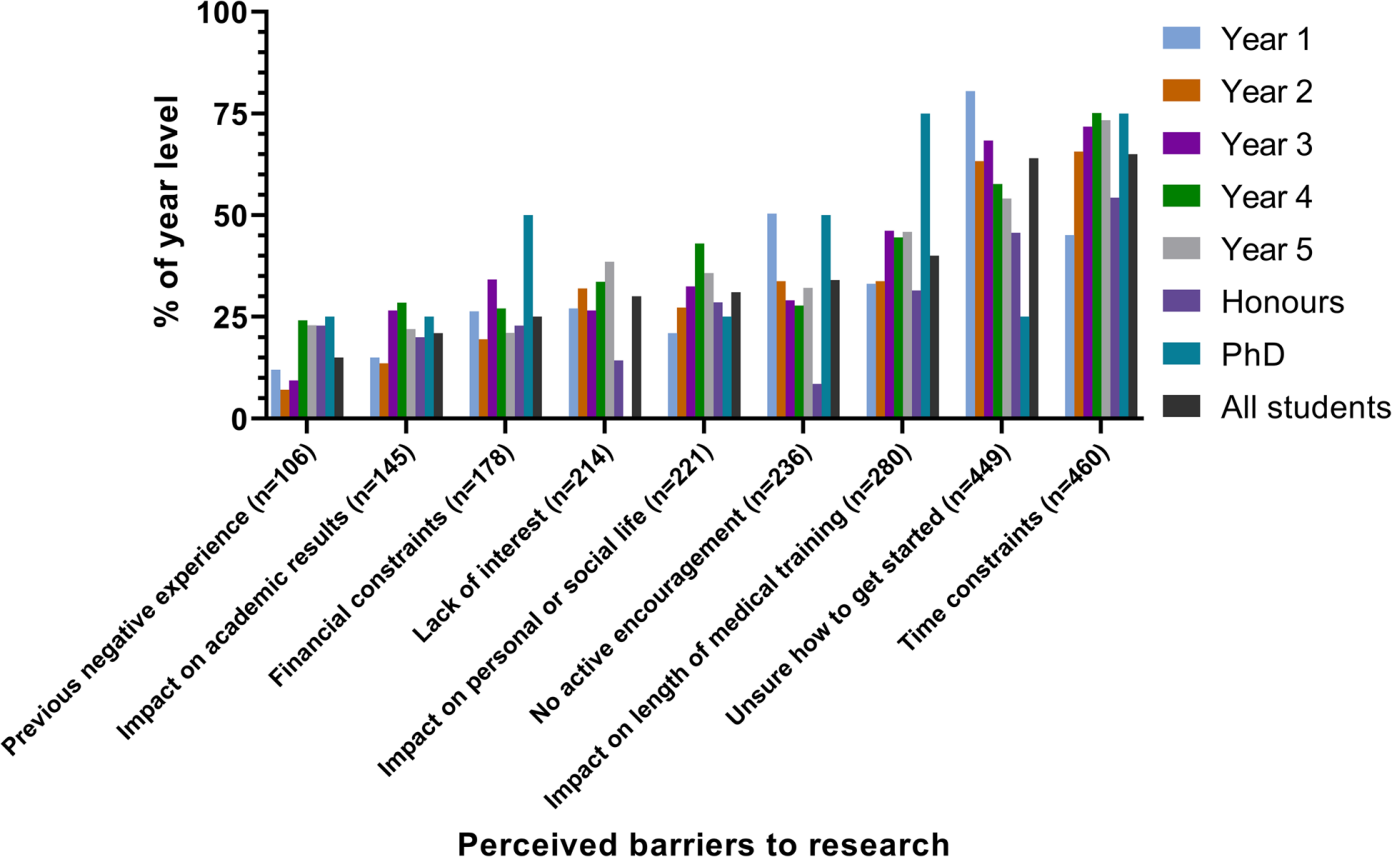
Perceived barriers to conducting research, stratified by year level

Barriers to research participation varied with year level (Fig. 2). Direct-entry students were more likely than graduate-entrants to cite “no active encouragement” (*n* = 30; 37.1% versus *n* = 206; 20.5%) and “unsure how to get started” (*n* = 59; 70.1% versus *n* = 390; 40.4%). Males were more likely to cite “impact on academic results” (*n* = 49; 26.3% versus *n* = 90; 20.7%).

### Understanding of research terms

Students were asked to self-assess their understanding of common types of research and research metrics.

Clinical research (*n* = 568; 81%) was the most well-known type of research out of clinical, epidemiological, basic, and translational research, while translational research (*n* = 141; 20%) was the least well-known. Seven percent of respondents were not familiar with any of the included types of research (*n* = 48). Graduate entry students were associated with greater familiarity with research types (χ^2^(1) = 3.899, *p* < 0.05). Understanding of types of research generally increased with both age and year level.

The majority of students of students (*n* = 375; 53%) were unaware of common research metrics. However, 280 students (40%) were familiar with Impact Factor and 57 students (8%) were familiar with H-Index. Graduate entry students were significantly more likely to be familiar with both Impact Factor (χ^2^(1) = 23.433, *p* < 0.001) and H-Index (χ^2^(1) = 5.985, *p* < 0.05). Understanding of research metrics generally increased with both age and year level.

### Aspirations for future research participation

**Fig. 3:**
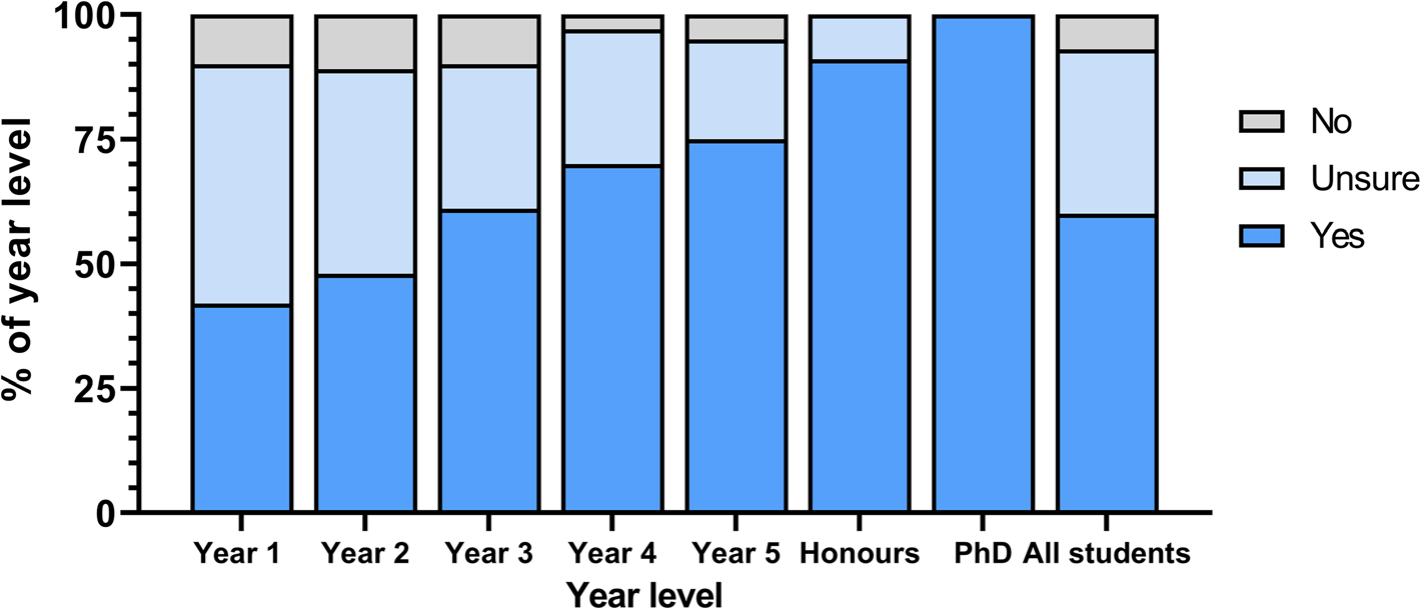
Student interest in pursuing future research, stratified by year level

The majority of students (*n* = 396; 59.9%) planned on pursuing future research; 217 students (32.6%) were unsure, and 50 students (7.5%) reported no interest. Desire to pursue research increased with advancing year level (see Fig. 3). Students interested in anaesthetics, physician, or surgical specialties were most likely to pursue further research (*p* > 0.05). Research interest did not vary between genders, nor between direct-entry and graduate-entry students (data not shown).

Of the 615 students potentially interested in pursuing research, 538 (87.5%) reported interest in clinical research, 319 (51.9%) in translational research, 305 (49.6%) in epidemiological research, 231 (37.6%) in basic research, and 167 (27.2%) in bioethical research. Note that students were able to select multiple responses. Interest in clinical and epidemiological research increased with advancing year level, while the inverse was true for basic and translational research.

Twenty-one percent (*n* = 148) of students stated a definite interest in pursuing an additional research degree; 49.7% (*n* = 349) were undecided and 29.3% (*n* = 205) were not interested. While this varied with year level (*p* < 0.001), type of enrolment and gender had no significant impact. Students interested in medical/physician specialities, surgical specialities, and anaesthetics were most likely to report interest, with only surgical specialities yielding a significant association (χ^2^(2) = 10.910, *p* < 0.01). Of the 148 students who reported definite interest, 106 (58.2%) were interested in the one-year intercalated BMedSc (Hons), while 63 (34.6%) reported interest in a PhD.

A moderate association existed between respondents interested in general practice and a lack of interest in pursuing both future research (χ2(2) = 44.244, *p* < 0.001) and an additional research degree (χ^2^(2) = 23.564, *p* < 0.001).

**Fig. 4:**
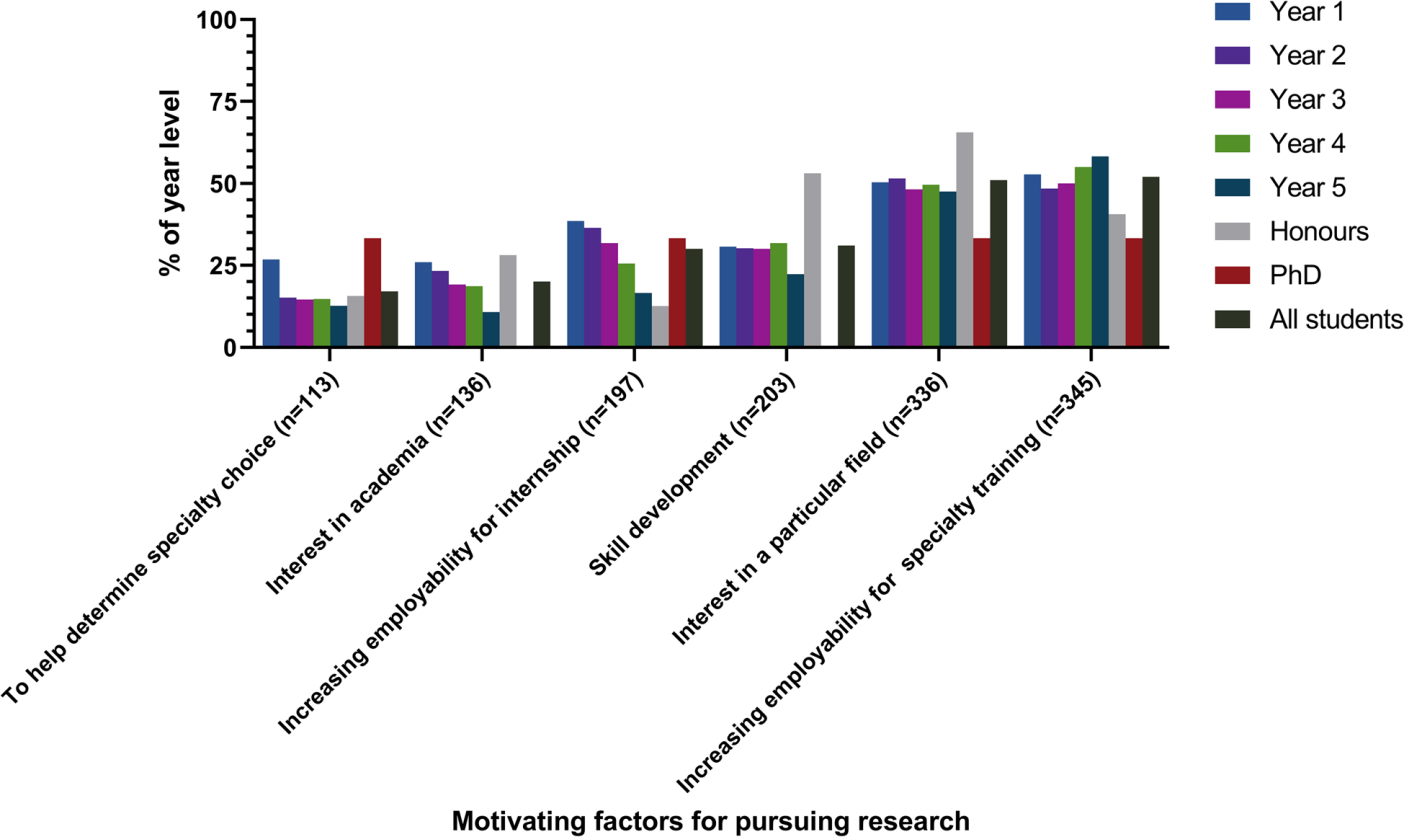
Motivating factors for pursuing research, stratified by year level

Students were asked to rate six factors on a scale ranging from “not important at all” to “very important” based on their influence on their motivation to undertake research. The factors that students deemed “very important” motivators are presented in Fig. 4.

### Student solutions to improving research participation

Three-hundred and thirty-two respondents (47.2% of the total study cohort) provided strategies to increase medical student research. Table 2 provides a thematic breakdown of free-text responses.

**Table 2:** Solutions offered by survey respondents regarding how to increase medical student research engagement

Themes	Representative responses	% of responses
**Improve access to and promotion of research opportunities**	“[Allow] earlier exposure to actual research as opposed to just teaching research methods”	51.8%
	“Updated databases of possible research project opportunities”	
	“A subscription service notifying students of new research opportunities tailored to the fields they want to pursue”	
	“More accessibility to full time researchers as clinicians are too busy”	
	“More opportunities for students in rural settings to do research”	
**Better education about both research skills and the importance of research**	“Faculty teaching … on research skills such as ethics submission and study design”	18.4%
	“Explaining the role of research in patient care”	
	“Knowing the weighting of research on internship and specialty applications”	
**Provide incentives to complete research**	“Better remuneration and appreciation, not treated as free labour with a $200/week summer research scholarship for fulltime work”	8.7%
	“Make it more interesting and financially accessible for students that cannot justify putting off getting paid for a whole year”	
**Allow dedicated time for research**	“Active exposure via shadowing researchers or Honours students”	6.9%
	“Further integration of research into the course such as [the programs at] Stanford and Harvard”	
**Actively encourage research**	“Active encouragement and platforms to showcase undergraduate research like [the International Conference of Undergraduate Research] and the research week”	3.6%
	“Consultants/medical staff that are associated with teaching hospitals [should be] encouraged to involve medical students in research projects”	
	“Active encouragement by faculty [with] tutors speaking about their own research projects”	
**Safeguard against the exploitation of medical students by researchers**	“Having increased accountability for supervisors to credit/acknowledge students as opposed to using them as guinea pigs for data collection”	2.1%
	“Better canvassing of opportunities with more opportunities to get involved where students are aware of time commitment and if [they will receive authorship]”	
**Publish stories about other medical student research experiences**	“Produce day-in-the-life stories/videos following BMedSc (Hons) students”	2.1%
	“Talks by current and past students about what [research is] really like, not just from the faculty”	
**Medical student research should** **not** **be promoted**	“Most BMedSc (Hons) projects are small and lacking in [academic or clinical] utility”	1.8%
	“Too many students do research [to increase employability] and are not actually interested in it”	
	“Doing research to get an internship/advanced training place produces non-contributory, [poor quality] research”	

## Discussion

This survey provides a detailed insight into the attitudes, enablers, and barriers to medical student research participation during medical school. Of note, from 2020, Monash University implemented a compulsory six-week scholarly intensive placement for final year medical students to work on a research, education, or professional practice project. This survey was administered in 2019 and thus reflects a cohort of respondents that had not undertaken this placement, although 39 (5.5%) respondents were undertaking an intercalated research degree at the time of data collection. Similarly, a small proportion of final year respondents had previously completed one or more years of formal research training.

Most medical students reported interest in pursuing research, which reflects previously reported data about Australian medical students [10, 12], but was slightly less than the research interest seen in medical students internationally [4]. The most important motivating factor was increasing employability for specialty training programs, even for students uncertain about their future specialty choice. Conversely, only a small number of students cited interest in academia as a motivator, a trend that decreased with advancing year level. Desire to enter more competitive specialties was additionally associated with greater research experience. This pragmatic approach to research participation is undoubtedly a global phenomenon [4]. This invariably reflects the competitiveness for entry into specialty training programs in Australia, with entry into some specialities so restricted that many applicants pursue additional research degrees prior to applying for training schemes. This may not be concerning in and of itself, but does validate that the pressure of entry to specialty training is a significant concern for many medical students. Within the framework of self-determination theory, the pursuit of research for the purpose of improving one’s employability can be considered a purely extrinsic aspiration and can therefore have implications for the student’s mental and physical wellbeing. As a result, programs should aim to promote the internalisation of such extrinsic aspirations in order to ensure that the students’ research pursuits are neither detrimental to themselves, nor unsustainable [13, 14]. Medical faculties should focus on espousing reasons to pursue research other than career advancement including the development of transferable skills, gaining a sense of shared achievement within a research group, and contribution to the improvement of patient care and outcomes. Rosenkranz et al. suggests a model by which students should be slowly exposed to research and research education over the duration of the medical course in order to promote internalisation of motivation. They suggest several practical strategies including scheduled research time, autonomy in selection of research projects, financial support for research participation, practical research education and support, and use of interpersonal relationships between students and their peers and/or research supervisors to foster a greater interest in research [11].

It should be noted that students who aim to apply for competitive training programs are more likely to be conscientious students, and therefore may be inclined to pursue research opportunities irrespective of their speciality choices. Nonetheless, there may be some merit in identifying and targeting students aiming for less competitive programs. Many specialities have student-run or professional college-sponsored interest groups that exist to promote that speciality to students and junior doctors; these may provide one avenue through which research education and opportunities can be provided to such students.

Interest in basic and translational research decreased with advancing year level, a trend which is consistent internationally and not unexpected as clinical exposure increases [4]. This finding should be considered when designing curricula, where more balanced exposure to different research types may be necessary. Integration of laboratory-based research into the clinical years may allow continued exposure to basic sciences, enhance understanding of translational research, and provide greater insight into the pathophysiological basis of disease and its treatment.

Research participation in this study (44.9%) was similar to that of a 2017 survey at the University of Queensland (60%) [10] but higher than that of a 2015 survey at the University of Western Sydney (7.4%), although this was conducted at a recently-established medical school with a smaller cohort of graduate-entry students [11]. The research participation rate was also slightly higher than the global medical student research participation rate of approximately one-third, reported in a 2015 meta-analysis of 79 studies [4]. Despite students in higher year levels having a greater breadth of research experience, over two-thirds of final year students still lacked experience in developing a research question, study design, submitting ethics applications, and recruiting participants. This is similar to data from the United Kingdom, where 59% of student research contributions were limited to data collection [15]. While any type of research participation is likely to provide benefit, exposure to aspects of research outside of data collection and entry likely allow for a deeper, more nuanced understanding of academic medicine. From a supervisorial perspective, researchers may actively encourage and support medical students to participate in the more challenging and complex aspects of a research project with appropriate training and supervision, especially given that students may be hesitant to ask to be involved with tasks they are unfamiliar with such as data analysis or manuscript preparation.

Of the students who reported research experience (*n* = 341; 44.9% of total study cohort), 40.9% (*n* = 121) received authorship for a research output, less than half of which had been published in a peer-reviewed journal. Roughly one-third of final year respondents had been named as an author on a peer-reviewed publication, significantly less than the 46.5% publication rate of final year students in the United States [16]. While the postgraduate model of tertiary education in the United States creates an older, more experienced, medical student cohort, the high publication rate in American medical schools may also be partly attributed to the high proportion of medical schools that offer MD-PhDs [9] and deliberate engagement strategies by faculties. Stanford University, for example, provides financial incentives for medical student research, including tuition fee subsidies and dedicated research funding [16]. The promotion of extracurricular research is particularly beneficial for students unable to pursue additional research degrees due to time or financial constraints. The Stanford model also requires that staff provide mentorship through research training and support to medical students as part of their faculty duties [10], a strategy that has shown promise in developing researchers [4, 16, 17]. However, it should also be noted that the competitive nature of the postgraduate model seen in the United States may also influence research participation due to the increased need for research experience in order to obtain a place in medical school and a residency match in a desired program. Conversely, research experience is not required for entry to Australian undergraduate medical programs, nor for some of its postgraduate programs, and therefore may not be prioritised by students.

The majority of students reported uncertainty around finding research opportunities. This reduced slightly over time but remained a factor for 54.1% (*n* = 59) of final year respondents. Consequently, a significant proportion of respondents suggested the need for a database listing research opportunities. While such a database exists at Monash University, it is vital that it is well-known to students to ensure its efficacy. Such a database should additionally be widely used by researchers, updated regularly, inclusive of short-term roles (e.g. data entry, ethics submission), and clearly define expected commitments and outcomes for students (e.g. acknowledgement, authorship, payment). A national, or even international, database may help link medical students with a greater breadth of research projects, while simultaneously bolstering the research workforce. A platform modelled on job seeking portals may be a viable option that would not be logistically complex to develop, and would allow students to ‘apply’ for projects they are interested in. Introducing students to research from the early years of the course, as well as explicit instructions for accessing research opportunities may additionally be warranted. Time constraints also appear to be a globally reported barrier to medical student research [4, 10]. Allocating dedicated time to conduct research throughout the academic year and integration of practical research into the curriculum could provide a workable solution. Notably, the only gender disparity seen in barriers to research was regarding the potential for research to impact on academic results, where a slightly greater proportion of males noted their concern. Interestingly, Amgad et al. reported no gender difference in research enablers and barriers [4]. Some studies, however, have identified that female medical students are more likely to be concerned about delays in training and its subsequent impact on child rearing [18, 19], although this was not evident in our survey.

A significant proportion of students were unaware of the different types of research, as well as what research entails. Additionally, the majority of students reported poor research literacy and a lack of familiarity with basic research metrics, the latter of which can be an indicator of familiarity with research and is important for interpretation of the literature. Although many curricula teach basic research skills, practical experience is critical for skill development. Extended research involvement over numerous years provides a pathway that allows for both continuing skill development and fluctuations in time commitment [1]. It is also vital that research education includes less widely taught skills, such as study design and manuscript writing, to ensure a more balanced development of research skills. It is likely that involvement in these aspects of the research process will foster a better understanding of the various types of research and improve overall research literacy.

Promoting extracurricular research or allowing dedicated time for research were popular suggestions amongst respondents. These are understandably difficult to implement, however, due to the delicate balance within medical curricula between research education and the clinical teaching required to produce proficient graduate doctors. Hence, increasing research education may come at the expense of clinical teaching and placement, or the duration of the medical degree. Given that the primary objective of medical school is to create safe, competent doctors, other solutions to promoting medical research beyond the medical school setting must also be considered. Eley suggests that a national, strategically-focussed approach to research training (e.g. a better-defined, uniform MD-PhD program with a standardised curriculum for research education) may provide students with a viable pathway for research [20]. A national approach to clinician-scientist training through an MD-PhD program may allow selected students to pursue research while simultaneously garnering clinical experience and without significantly extending their degree, although it should be noted that this pathway can be challenging and arduous and is therefore only appropriate for a small minority of medical students. At the junior doctor and speciality trainee level, continuing research education and promotion of research opportunities alongside clinical training may be warranted. Phang et al. reports that junior doctors typically struggle to access such opportunities due to their clinical workload and the expectation that research be conducted outside of paid hours, along with a lack of accessible avenues to engage with research [21]. While some training programs mandate a ‘Scholarly Project’ as part of their summative assessments, more health services could consider integrating research training within their programs in order to bolster research engagement by their junior staff. On a larger scale, Australia spent 1.8% of its gross domestic product (GDP) in 2017 on research funding, well below the average amongst Organisation for Economic Co-operation and Development (OECD) countries [22], indicating a need for much greater investment into research in general.

A limitation of this study is the recruitment from a single institution, although the study cohort is both large and diverse, representing one-third of the entire Monash University medical student population. There is also potential for self-selection bias due to the voluntary nature of the survey, which is likely to over-represent those interested in research. The creation of a new survey, as opposed to the use of a validated tool, similarly presents a limitation in the collection of data. However, this was intentional; the survey was created combining elements from multiple surveys to better suit an Australian medical student cohort.

It is intended to extend this survey to other Australian medical schools, allowing analysis of inter-university differences, and to confirm the external validity of the results. With the recent introduction of the MD to many Australian universities, further assessment of medical student research attitudes and practices will be necessary in order to track changes that occur as a result of more research-oriented programs.

## Conclusion

Students at Australia’s largest medical school have a high desire for, but limited experience with research, and are primarily motivated by the prospect of increasing employability for specialist training. A number of approaches to promoting medical student research exist, and should be implemented, but ultimately should focus on fostering intrinsic motivation to pursue research. A greater focus on research education and promotion within medical school curricula will also be valuable in bolstering the research involvement of future clinicians, developing the skills necessary to practice evidence-based medicine, and providing the benefits of research training to all medical graduates.

## Supplementary Information

**Additional file 1.** Survey questions. Description of data: Questions included in the Qualtrics XM® survey distributed to study participants.

## Data Availability

The datasets used and/or analysed during the current study are available from the corresponding author on reasonable request.

## Abbreviations

BMedSc (Hons): Bachelor of Medical Science (Honours)
GDP: Gross domestic product
MD-PhD: Doctor of Medicine/Doctor of Philosophy
OECD: Organisation for Economic Co-operation and Development
PhD: Doctor of Philosophy

## Footnotes

1 While most reported research experiences are likely to have been from during medical school, for the small proportion of graduate-entry students, these experiences may have occurred during their undergraduate degree.

## References

[1] Frishman WH (2001). Student research projects and theses: should they be a requirement for medical school graduation?. Heart Dis.

[2] Hunskaar S, Breivik J, Siebke M, Tommeras K, Figenschau K, Hansen JB (2009). Evaluation of the medical student research programme in Norwegian medical schools. A survey of students and supervisors. BMC Med Educ.

[3] Jain MK, Cheung VG, Utz PJ, Kobilka BK, Yamada T, Lefkowitz R (2019). Saving the endangered physician-scientist - a plan for accelerating medical breakthroughs. N Engl J Med.

[4] Amgad M, Man Kin Tsui M, Liptrott SJ, Shash E (2015). Medical student research: an integrated mixed-methods systematic review and meta-analysis. PLoS One.

[5] Hyde S (2007). Australian medical students’ interest in research as a career. Focus Health Prof Educ.

[6] Reinders JJ, Kropmans TJ, Cohen-Schotanus J (2005). Extracurricular research experience of medical students and their scientific output after graduation. Med Educ.

[7] Brass LF (2018). Is an MD/PhD program right for me? Advice on becoming a physician-scientist. Mol Biol Cell.

[8] Eley DS, Benham H (2016). From medical student to clinician-scientist: where is the pathway in Australia?. Intern Med J.

[9] Mileder LP (2014). Medical students and research: Is there a current discrepancy between education and demands?. GMS Z Med Ausbild.

[10] Eley DS, Jensen C, Thomas R, Benham H (2017). What will it take? Pathways, time and funding: Australian medical students' perspective on clinician-scientist training. BMC Med Educ..

[11] Rosenkranz SK, Wang S, Hu W (2015). Motivating medical students to do research: a mixed methods study using self-determination theory. BMC Med Educ.

[12] Medical Schools Outcomes Database (2020). National data report 2020. Medical Deans Australia and New Zealand.

[13] Kasser T, Ryan RM (1996). Further examining the American dream: differential correlates of intrinsic and extrinsic goals. Personal Soc Psychol Bull.

[14] Patrick H, Williams GC (2012). Self-determination theory: its application to health behavior and complementarity with motivational interviewing. Int J Behav Nutr Phys Act.

[15] Griffin MF, Hindocha S (2011). Publication practices of medical students at British medical schools: experience, attitudes and barriers to publish. Med Teach.

[16] Jacobs CD, Cross PC (1995). The value of medical student research: the experience at Stanford University School of Medicine. Med Educ.

[17] Reynolds HY (2008). In choosing a research health career, mentoring is essential. Lung..

[18] Boulis AK, Jacobs JA (2011). The changing face of medicine: women doctors and the evolution of health care in America: Cornell University press.

[19] Shollen SL, Bland CJ, Finstad DA, Taylor AL (2009). Organizational climate and family life: how these factors affect the status of women faculty at one medical school. Acad Med.

[20] Eley DS (2018). The clinician-scientist track: an approach addressing Australia's need for a pathway to train its future clinical academic workforce. BMC Med Educ..

[21] Phang DTY, Rogers GD, Hashem F, Sharma S, Noble C. Factors influencing junior doctor workplace engagement in research: an Australian study. Focus Health Prof Educ. 2020;21(1). 10.11157/fohpe.v21i1.299.

[22] OECD (2020). Gross domestic spending on R&D (indicator).

