# Supplementary material for: The experiences, attitudes and understanding of research amongst medical students at an Australian medical school
Source: BMC Med Educ. 2021 May 10;21:267. doi: 10.1186/s12909-021-02713-9 (PMC8108334; doi:10.1186/s12909-021-02713-9)
Supplement: Supplementary file 1 — Additional file 1. Survey questions. Description of data: Questions included in the Qualtrics XM® survey distributed to study participants. [file 12909_2021_2713_MOESM1_ESM.docx]

**The experiences, attitudes and understanding of research amongst medical students at an Australian medical school**

**Authors:**

Jaidyn Muhandiramge^a^* (Co-first author)

Tony Vu^a^ (Co-first author)

Megan J. Wallace^b, c^

Eva Segelov^a, d^

^a^ Faculty of Medicine, Nursing and Health Sciences, Monash University, Melbourne, Australia

^b^ Department of Obstetrics and Gynaecology, Monash University, Melbourne, Australia

^c^ The Ritchie Centre, Hudson Institute of Medical Research, Melbourne, Australia

^d^ Department of Medical Oncology, Monash Health, Melbourne, Australia

**Corresponding author:**

Jaidyn Muhandiramge

[jaidyn.muhandiramge@monash.edu](mailto:jaidyn.muhandiramge@monash.edu)

Faculty of Medicine, Nursing and Health Sciences, Monash University

27 Rainforest Walk, Clayton, Victoria, Australia 3800

Phone: +61 3 8572 2392

**Additional file 1: Survey questions**

**Age**

18-21

22-25

26-29

30+

**Gender**

Male

Female

Other

Prefer not to say

**Current enrolment**

Undergraduate

Postgraduate

**Year level**

Year 1

Year 2A

Year 3B

Year 4C

Year 5D

Bachelor of Medical Science (Honours)

PhD

**Type of medical place**

Commonwealth Supported Place (CSP)

Bonded Medical Place (BMP)

Extended Rural Cohort (ERC)

Full Fee Paying (FFP)

**Do you plan on pursuing an additional research degree?**

Yes

Undecided

No

*If “Yes” or “Undecided”:*

What type of additional research degree do you plan on pursuing? Please select all that apply.

Bachelor of Medical Science (Honours)

PhD

Other (please specify) ________________________________________________

**What specialties are you most likely to pursue? Please select all that apply.**

Unsure

Medical/Physician

Paediatrics

Surgical

Obstetrics & Gynaecology

Anaesthetics

Psychiatry

General Practice

Other (please specify) ________________________________________________

**Which of the following terms are you familiar with? Please select all that apply.**

Basic Research

Clinical Research

Translational Research

Epidemiological Research

Bioethical Research

None of the above

**Which of the following terms are you familiar with? Please select all that apply.**

Impact factor

h-index

None of the above

**How important do you think research is for:**

|  | Not Important At All | Not Very Important | Neutral | Important | Very Important |
| --- | --- | --- | --- | --- | --- |
| Applying for an intern position |  |  |  |  |  |
| Applying for a specialty training program |  |  |  |  |  |

**Do you see yourself being involved in research in your future career?**

Yes

Unsure

No

*If “Yes” OR “Unsure”:*

**What areas of research are you interested in? Please select all that apply.**

Basic (e.g. genetic studies, laboratory research)

Translational i.e. the use of basic scientific research to create new medical therapies or diagnostic procedures

Clinical (e.g. clinical trials, prognostic studies, case reports)

Epidemiological/Health service (e.g. cohort studies, case control studies)

Bioethical research i.e. the study of ethical, social and legal issues that arise in medicine and medical research

Other (please specify) ________________________________________________

**How important are the following factors in motivating you to pursue research opportunities?**

|  | Not Important At All | Not Very Important | Neutral | Important | Very Important |
| --- | --- | --- | --- | --- | --- |
| Interest in understanding the basic processes of research |  |  |  |  |  |
| Interest in a particular field |  |  |  |  |  |
| Interest in academia |  |  |  |  |  |
| Skill development to set yourself up for future research |  |  |  |  |  |
| To help determine which specialty you want to pursue |  |  |  |  |  |
| Increasing employability for intern year |  |  |  |  |  |
| Increasing employability for a specialty training program |  |  |  |  |  |

**How many research projects have you been involved with in any capacity?**

0

1-3

4-6

7-9

10+

*If ≥”1-3”:*

**When did you first get involved with research?**

Previous degree

Pre-clinical years

Clinical years

Bachelor of Medical Science (Honours)

Other (please specify) ________________________________________________

**How positive have your research experiences been overall on a scale of 1 (extremely negative) to 5 (extremely positive)?**

**What contributions have you made to a research project? Please select all that apply.**

Developing a research question

Conducting a literature review

Designing a study

Submitting an ethics application

Recruiting participants

Collecting data

Inputting/entering data

Analysing data

Preparing a presentation or manuscript

**Have you been named as an author as a result of a research project?**

Yes

No

*If “Yes”:*

**What have been the outcomes of your research experiences? Please select all that apply.**

Presented at student conferences

Presented at local meetings/conferences

Presented at national conferences

Presented at international conferences

Published in a peer-reviewed journal

No outcome/project still ongoing

Other (please specify) ________________________________________________

*If “No”:*

**What have been the outcomes of your research experiences? Please select all that apply.**

Presented at student conferences

Presented at local meetings/conferences

Presented at national conferences

Presented at international conferences

Published in a peer-reviewed journal

No outcome/project still ongoing

Other (please specify) ________________________________________________

**What factors have affected your participation in research? Please select all that apply.**

Lack of interest

Time constraints

Impact on personal or social life

Impact on length of medical training

Financial constraints

Impact on academic results

Unsure how to get started

Previous experience with research

No active encouragement

Other (please specify) ________________________________________________

**What strategies do you think would promote medical student research?**

[Free text]
